# von Hippel-Lindau Disease-Associated Hemangioblastomas Are Derived from Embryologic Multipotent Cells

**DOI:** 10.1371/journal.pmed.0040060

**Published:** 2007-02-13

**Authors:** Deric M Park, Zhengping Zhuang, Ling Chen, Nicholas Szerlip, Irina Maric, Jie Li, Taesung Sohn, Stephanie H Kim, Irina A Lubensky, Alexander O Vortmeyer, Griffin P Rodgers, Edward H Oldfield, Russell R Lonser

**Affiliations:** 1 Surgical Neurology Branch, National Institute of Neurological Disorders and Stroke, National Institutes of Health, Bethesda, Maryland, United States of America; 2 Molecular and Clinical Hematology Branch, National Institute of Diabetes and Digestive and Kidney Disease, National Institutes of Health, Bethesda, Maryland, United States of America; 3 Hematology Section, Department of Laboratory Medicine, Warren G. Magnuson Clinical Center, National Institutes of Health, Bethesda, Maryland, United States of America; Brain Tumor Institute, United States of America

## Abstract

**Background:**

To determine the origin of the neoplastic cell in central nervous system (CNS) hemangioblastomas in von Hippel-Lindau disease (VHL) and its role in tumor formation and distribution, we characterized and differentiated neoplastic cells from hemangioblastomas removed from VHL patients.

**Methods and Findings:**

A total of 31 CNS hemangioblastomas from 25 VHL patients were resected and analyzed. Tumor cells from the hemangioblastomas were characterized, grown, and differentiated into multiple lineages. Resected hemangioblastomas were located in the cerebellum (11 tumors), brainstem (five tumors), and spinal cord (15 tumors). Consistent with an embryologically derived hemangioblast, the neoplastic cells demonstrated coexpression of the mesodermal markers brachyury, Flk-1 (vascular endothelial growth factor-2), and stem cell leukemia (Scl). The neoplastic cells also expressed hematopoietic stem cell antigens and receptors including CD133, CD34, c-kit, Scl, erythropoietin, and erythropoietin receptor. Under specific microenvironments, neoplastic cells (hemangioblasts) were expanded and differentiated into erythrocytic, granulocytic, and endothelial progenitors. Deletion of the wild-type *VHL* allele in the hematopoietic and endothelial progeny confirmed their neoplastic origin.

**Conclusions:**

The neoplastic cell of origin for CNS hemangioblastomas in VHL patients is the mesoderm-derived, embryologically arrested hemangioblast. The hematopoietic and endothelial differentiation potential of these cells can be reactivated under suitable conditions. These findings may also explain the unique tissue distribution of tumor involvement.

## Abstract 2

In this study, the neoplastic cell of origin for central nervous system hemangioblastomas in patients with von Hippel-Lindau disease is shown to be the mesoderm-derived, embryologically arrested hemangioblast.

## Editors' Summary

**Background.:**

von Hippel-Lindau (VHL) disease is a rare genetic condition characterized by the development of benign and malignant tumors in multiple organ systems. All the cells of people with this disorder contain one normal copy of the *VHL* gene and one altered copy. This gene encodes a tumor suppressor, a protein that stops tumors growing. One functioning copy of the *VHL* gene is sufficient to prevent any problems, but if the remaining normal copy becomes altered (mutated) in individual cells of patients with VHL disease, tumors result. These tumors are mainly benign (noncancerous growths that do not spread around the body) and form in parts of the body that are rich in blood vessels, in particular in the retina (the back of the eye), the cerebellum (the back of the brain), the brainstem (which links the cerebellum to the spinal cord), and the spinal cord. These central nervous system (CNS) tumors are called hemangioblastomas and look like little knots of capillaries (fine blood vessels). As they grow, they can cause problems through fluid leakage or by pressing on brain tissue. There is no cure for VHL disease, but patients can be monitored and their tumors dealt with before they get too large.

**Why Was This Study Done?:**

It is not known what sort of cells hemangioblastomas develop from or why they occur only in specific parts of the CNS. This information could help researchers develop ways to prevent or treat these hemangioblastomas. One possibility is that hemangioblastomas develop from a special kind of embryonic cell called a hemangioblast. This multipotent stem cell—a continuously dividing cell that can develop (differentiate) into several nondividing cell types—is the source of blood cells and blood vessel cells in the embryo. In this study, the researchers have examined tumor cells (so-called neoplastic stromal cells) taken from hemangioblastomas to see whether this theory is correct.

**What Did the Researchers Do and Find?:**

The researchers obtained several CNS hemangioblastomas from patients with VHL disease and stained slices of them with antibodies that stick to proteins made only by specific types of embryonic cells. This experiment showed that the neoplastic stromal cells in the tumors contained two proteins (brachyury and Flk-1) that mark hemangioblast cells in embryos and a protein called Scl that is required for blood cell formation. The neoplastic stromal cells also made several proteins expressed by the precursors of different blood cell types. When the researchers grew neoplastic stromal cells from the hemangioblastomas in different conditions, they found that the cells differentiated into the precursors of two types of blood cell (erythrocytes and granulocytes) and of the cells that line blood vessels (endothelial cells). The researchers confirmed that these precursors had arisen from the neoplastic cells in the hemangioblastomas by showing that they did not contain a normal copy of the *VHL* gene.

**What Do These Findings Mean?:**

These findings indicate that the neoplastic stromal cells in CNS hemangioblastomas in patients with VHL disease are indeed hemangioblasts. Their expression of the protein brachyury, which is normally only expressed in early development, suggests that these hemangioblasts were arrested during embryogenesis, possibly when they lost their normal *VHL* gene. Because VHL-related tumors usually appear in adulthood, circulating factors such as hormones might promote the growth of these pre-existing but dormant tumor cells at specific stages of life. Furthermore, the expression of Scl in the neoplastic stromal cells suggests that the specific pattern of hemangioblastomas in VHL disease is determined during development since Scl is transiently expressed in hemangioblasts in the parts of the developing brain that subsequently harbor hemangioblastomas. Because the researchers only examined VHL-associated hemangioblastomas, their findings do not explain the origin of hemangioblastomas in patients without VHL disease or the origin of VHL-associated malignant tumors. However, the identification of hemangioblasts as the source of hemangioblastomas in VHL disease suggests that it might be possible to treat these tumors by persuading the hemangioblasts to differentiate or by using their unique proteins to target toxic drugs to hemangioblastomas.

**Additional Information.:**

Please access these Web sites via the online version of this summary at http://dx.doi.org/10.1371/journal.pmed.0040060.

- VHL Family Alliance has information for patients and physicians on VHL disease
- The US National Institute of Neurological Disorders and Stroke Web site contains information on VHL disease
- Wikipedia carries pages on VHL disease (note that Wikipedia is a free online encyclopedia that anyone can edit)
- The Online Mendelian Inheritance in Man (OMIM) site has detailed scientific information on VHL disease

## Introduction

von Hippel-Lindau disease (VHL) is an autosomal dominant neoplasia syndrome resulting from a germline mutation in the *VHL* tumor suppressor gene on the short arm of Chromosome 3 [1]. VHL has an approximate prevalence of one in 39,000 and over 90% penetrance by 65 years of age [2,3]. Germline mutations in the *VHL* gene lead to the development of a number of benign or malignant tumors and cysts in multiple organ systems [4]. Affected individuals may develop central nervous system (CNS) tumors, including hemangioblastomas and endolymphatic sac tumors [4–7]. Visceral manifestations include renal cysts and carcinomas, pheochromocytomas, pancreatic cysts, and neuroendocrine tumors, as well as epididymal and broad ligament cystadenomas [4,8–10].

CNS hemangioblastomas are the most common tumor in VHL, affecting 60%–80% of all patients, and are a frequent cause of neurologic morbidity and mortality [4,7,11]. Hemangioblastomas occur in highly defined regions of the CNS that include the retina, cerebellum, brainstem, and spinal cord [4,7,11]. Histologically, hemangioblastomas are benign, highly vascular tumors composed of neoplastic stromal cells and vessels. Detailed morphologic analyses demonstrate intratumoral blood island formation indicative of extramedullary hematopoiesis [12,13].

Although the anatomic distribution and histologic features of hemangioblastomas in VHL suggest they may be derived from a developmental multipotent cell, the identity and function of the cell of origin in hemangioblastomas remains unknown. To determine the tumor cell of origin, the basis for the unique CNS distribution of hemangioblastomas, and the multipotent capability of hemangioblastomas in VHL, we analyzed these tumors for embryologic and hematopoietic stem cell markers. Based on the characterized features of these tumors, we then derived hematopoietic and endothelial progeny by microenvironment manipulation of resected hemangioblastoma neoplastic cells.

## Methods

### Patients

Twenty-five VHL patients who underwent resection of a CNS hemangioblastoma(s) between 2003 and 2005 at the NIH were included. Patients were screened for the presence of mutations and deletions of the *VHL* gene. All 25 patients met the diagnostic criteria for VHL [4]. Patients underwent serial craniospinal magnetic resonance imaging (MRI), and the location of all imaged hemangioblastomas was recorded. Research was performed under an institutional review board-approved protocol (National Institute of Neurological Disorders protocol 03-N-0164) and informed consent was obtained from all patients.

### Characterization of Hemangioblastomas

Frozen tumor sections were fixed in methanol, washed in PBS, and blocked with 5% serum (same species as the secondary antibody) containing 0.2% Triton X-100. Slides were stained with various antibodies overnight at 4 °C. Staining antibodies included brachyury (10 μg/ml; R&D Systems, http://www.rndsystems.com), Flk-1 (vascular endothelial growth factor [VEGF] receptor-2) (15 μg/ml; R&D Systems), stem cell leukemia (Scl) (1:100; Santa Cruz Biotechnology, http://www.scbt.com), CD133 (1:10; Miltenyi Biotec, http://www.miltenyibiotec.com), CD34 (1:100; Santa Cruz Biotechnology), stem cell factor receptor (c-kit) (1:500; DakoCytomation, http://www.dako.com), interleukin (IL)-3 receptor (1:50; Sigma, http://www.sigmaaldrich.com), erythropoietin (1:200; Sigma), and erythropoietin receptor (1:100; Sigma). After washing in PBS, primary antibodies were detected with AlexaFluor 488 and 555 secondary antibodies (1:2,000; Molecular Probes, http://probes.invitrogen.com). 4′,6-diamidino-2-phenylindole (DAPI) (300 nM; Molecular Probes) was used for nuclear counterstaining. Incubation of secondary antibodies without primary antibodies served as negative controls.

To confirm immunocytochemical findings, Western blot analyses were performed. Protein extraction of hemangioblastoma tissue was performed by three cycles of freezing and thawing of tumor tissue. Extracted protein was subjected to 4%–20% SDS–polyacrylamide gel (Invitrogen, http://www.invitrogen.com) electrophoresis, transferred to nitrocellulose membrane (Invitrogen), probed with brachyury antibody (0.2 μg/ml; R&D Systems), Scl antibody (1:200; Active Motif, http://www.activemotif.com), Flk-1 (VEGF receptor-2) antibody (0.2 μg/ml; R&D Systems) and alpha-tubulin (positive control at 1:1,000; Sigma). Species-specific horseradish peroxidase-conjugated secondary antibody (1:30,000; Upstate, http://www.upstate.com) was detected by enhanced chemiluminescence substrate (Pierce Biotechnology, http://www.piercenet.com).

### Cell Culture

Central cores of tumor from freshly resected VHL-associated hemangioblastomas were excised. These cores were rinsed in Hank's balanced salt solution (Cambrex, http://www.cambrex.com) and immediately placed in growth medium (see description below). Single-cell suspensions of tumors were established by manual trituration, enzymatic digestion, and mechanical disaggregation (MediMachine; Becton Dickinson, http://www.bdbiosciences.com). Contaminating red blood cells were removed by ACK cell lysing buffer (Cambrex) and density-gradient centrifugation. The identity of the isolated cells in suspension was determined by analyzing aliquots for characteristic well-described morphology, hematoxylin and eosin staining, Giemsa staining, immunocytochemistry, and/or loss of heterozygosity analyses. Cells identified as hemangioblastoma tumor cells were placed in wells coated with poly-L-ornithine (495 μg/ml; Sigma) and fibronectin (10 μg/ml; R&D Systems). The wells contained alpha-MEM (Sigma) or RPMI (Gibco, http://www.invitrogen.com) and were supplemented with fetal bovine serum (30%; Cambrex), BSA (10%), beta-mercaptoethanol (0.1 μmol/l), L-glutamine (1.5 mmol/l), penicillin (100 U/ml), streptomycin (100 μg/ml), transferrin (120 μg/ml), selenium (6.7 ng/ml; Gibco), insulin (10 μg/ml; Gibco), ferrous sulfate (900 ng/ml), ferric nitrate (90 ng/ml), dexamethasone (0.01 μmol/l), stem cell factor (100 ng/ml; R&D Systems) and erythropoietin (50–100 U/ml; R&D Systems). Fresh medium was added every 3 d. The tumor cells were resuspended and counted with a hemacytometer every 3 d.

### Differentiation of Hemangioblasts

#### Hematopoietic cells.

Tumor cells were placed in plates and dishes coated with poly-L-ornithine (495 μg/ml; Sigma) and fibronectin (10 μg/ml; R&D Systems). The cells were placed in culture containing alpha-MEM (Sigma) and RPMI (Gibco) supplemented with fetal bovine serum (30%; Cambrex), BSA (10%), beta-mercaptoethanol (0.1 μmol/l), L-glutamine (1.5 mmol/l), penicillin (100 U/ml), streptomycin (100 μg/ml), transferrin (120 μg/ml), selenium (6.7 ng/ml, Gibco), insulin (10 μg/ml; Gibco), ferrous sulfate (900 ng/ml), ferric nitrate (90 ng/ml), insulin (10 μg/ml), dexamethasone (0.01 μmol/l), stem cell factor (100 ng/ml; R&D Systems), erythropoietin (100 U/ml; R&D Systems), IL-3 (10 ng/ml; R&D Systems) and GM-CSF (10 ng/ml; R&D Systems), with and without G-CSF (50 ng/ml; R&D Systems). Fresh medium was added every 3 d. Nonadherent cells were resuspended and counted with a hemacytometer every 3 d.

#### Endothelial cells.

Tumor cells were placed in plates and dishes coated with poly-L-ornithine (495 μg/ml; Sigma) and fibronectin (10 μg/ml; R&D Systems). The cells were placed in culture containing IMDM (Gibco), fetal bovine serum (30%; Cambrex), BSA (10%), L-glutamine (1.5 mmol/l), penicillin (100 U/ml), streptomycin (100 μg/ml), transferrin (120 μg/ml), selenium (6.7 ng/ml; Gibco), insulin (10 μg/ml, Gibco), VEGF (5 ng/ml; R&D Systems), stem cell factor (100 ng/ml; R&D Systems), IGF-1 (10 ng/ml; R&D Systems), fibroblast growth factor-2 (10 ng/ml; R&D Systems), IL-11 (50 ng/ml; R&D Systems), erythropoietin (15 U/ml, R&D Systems) and endothelial cell growth supplement (100 μg/ml; Upstate). Fresh medium was added every 3 d.

### Nested Reverse Transcription Polymerase Chain Reaction

Cells morphologically consistent with a myeloid origin were analyzed for the expression of CD13, a myeloid marker, by nested reverse transcription PCR (RT-PCR) [14]. Briefly, total RNA was extracted and reverse-transcribed and amplified by two rounds of PCR. Product of the second-round PCR was electrophoresed on agarose gel and stained with ethidium bromide.

### Loss of Heterozygosity Analysis

Investigated tissues included uncultured tumor cells, cultured tumor cells, cultured nucleated red blood cells, cultured immature white blood cells, and cultured endothelial cells. Peripheral blood-derived mononuclear cells from patients served as a negative control. Specific cell types (identified morphologically and immunohistochemically; as described in text) were individually microdissected from slides for DNA extraction [15], and analyzed by PCR for loss of heterozygosity using the microsatellite markers D3S1038 and D3S1110 (Invitrogen) that flank the VHL gene [13]. The samples were electrophoresed on a polyacrylamide gel for detection of the wild-type VHL allele.

### Characterization of Cultured Cells

Hemangioblastoma tumor cells were harvested and applied onto poly-D-lysine–coated slides by cytospin. Erythropoietin and erythropoietin receptor immunostaining was performed on tumor cells, as described above. Giemsa staining was performed using modified Giemsa solution (1:10; Sigma). Adherent cells were costained with Tie-2 (10 μg/ml; R&D Systems) and CD31/PECAM-1 (5 μg/ml; R&D Systems) as described above.

## Results

### Patient Characteristics

**Figure 1:**
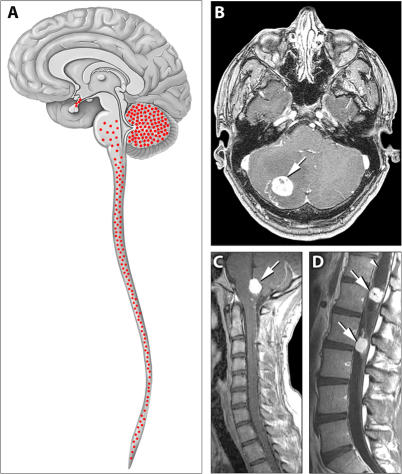
Distribution of Hemangioblastomas in the Central Nervous Systems of Study Patients (A) Schematic representation of the distribution of CNS hemangioblastomas (red dots) in the 25 von Hippel-Lindau disease patients on MRI. Most (98%) of hemangioblastomas were found below the level of the tentorium in the cerebellum, brainstem, and spinal cord. (B–D) Contrast-enhanced MRI demonstrating representative locations of hemangioblastomas including the cerebellum (B), brainstem (C) and spinal cord (D). (B) Axial view through the cerebellum demonstrating a hyperintense enhancing hemangioblastoma (arrow) with surrounding edema (hypointense area surrounding the tumor) that frequently is associated with these lesions. (C) Sagittal view through the posterior fossa demonstrating a hyperintense enhancing brainstem (medullary) hemangioblastoma (arrow) with surrounding edema. (D) Sagittal view through the thoracic and lumbar spinal cord demonstrating two hyperintense enhancing hemangioblastomas (arrows). The superior tumor is associated with a large intraspinal cyst (syrinx) that is common with these neoplasms (arrowhead).

Twenty-five VHL patients (13 females, 12 males) underwent resection of 31 CNS hemangioblastomas. Mean age at surgery was 34.9 ± 10.0 y (range 18–51 y). All patients had germline mutations of the *VHL* gene. MRI revealed 244 craniospinal hemangioblastomas distributed in the cerebellum (111 tumors; 45%), brainstem (14 tumors; 6%), and spinal cord (115 tumors; 47%). There were four (2%) supratentorial hemangioblastomas in the region of the pituitary stalk (three tumors) and gyrus rectus (one tumor). Hemangioblastomas that were removed and studied were from the cerebellum (11 tumors; 35% of tumors), brainstem (five tumors; 16%), or spinal cord (15 tumors; 48%) (Figure 1).

### Identification of Tumor Cell of Origin

**Figure 2:**
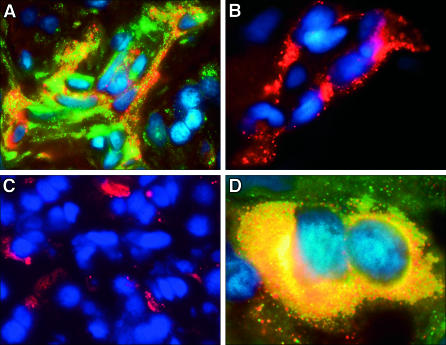
Immunofluorescent Characterization of the Neoplastic Stromal Cells in Hemangioblastoma Tissue Sections (A) Neoplastic cells express the mesodermal marker, brachyury (green), Flk-1 (VEGF receptor-2; red), and the nuclear stain 4′,6-diamidino-2-phenylindole (DAPI) (blue). Coexpression of brachyury and Flk-1 (VEGF receptor-2) indicates that these cells are embryologic hemangioblasts. (B) Expression of Scl (red) by the neoplastic cells indicates that these are hematopoietic stem cells committed to hematopoietic and endothelial lineages. (C) The neoplastic cells demonstrate scattered cytoplasmic expression of the hematopoietic stem cell antigen CD133 (red). (D) Coexpression of the erythropoietin (green) and erythropoietin receptor (red) was uniformly demonstrated on the neoplastic cells.

**Figure 3:**
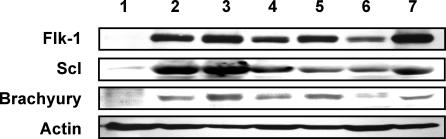
Western Blot Analysis Confirms Expression of Hemangioblast Markers in Tumor Cells Tumor samples (lanes 2 through 7) revealed expression of Flk-1 (VEGF receptor-2), Scl, and brachyury. Control (lane 1; normal brain) did not show expression of these proteins.

All tumors were histologically confirmed to be hemangioblastomas by routine staining. Immunofluorescence microscopy demonstrated that the hemangioblastoma neoplastic stromal cells expressed the mesodermal marker brachyury. Brachyury, a founding member of the T-box family of transcription factors, has a conserved role in mesoderm differentiation in vertebrates. Developmental studies indicate that early specification of posterior mesoderm and formation of the notochord is regulated by brachyury [16]. Coexpression of brachyury and Flk-1 (VEGF receptor-2) confirmed that the neoplastic cells were derived from the hemangioblast subset of mesodermal cells (Figure 2). Flk-1 (VEGF receptor-2) is a type 3 receptor tyrosine kinase that is believed to play a critical role in developmental angiogenesis and hematopoiesis [17]. Expression of Scl, which is essential for both primitive and definitive hematopoiesis, was demonstrated in the tumors (Figure 2). Expression of these embryologic markers was confirmed by Western blot analysis (Figure 3).

### Cellular Markers

To characterize the multipotent capacity of the hemangioblastoma neoplastic cells, we looked for expression of known stem cell markers. Immunofluorescence demonstrated expression of the stem cell marker CD133 (Figure 2C) and the hematopoietic stem cell marker CD34 in a few scattered cells throughout the hemangioblastomas. Similarly, c-kit and the common myeloid antigen, IL-3 receptor, were expressed in tumor cells in scattered regions throughout the hemangioblastomas. Coexpression of erythropoietin and erythropoietin receptor was demonstrated throughout the tumors (Figure 2D).

### Primary Culture of Neoplastic Cells

**Figure 4:**
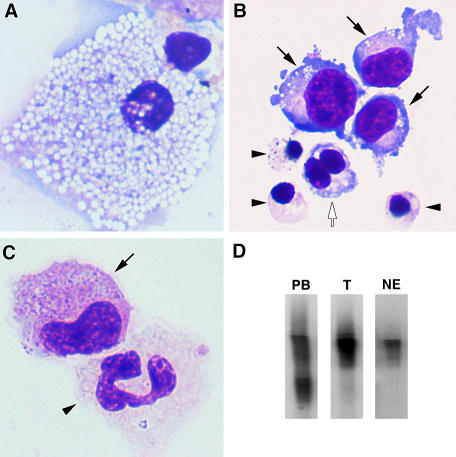
Hematopoietic Progenies Are Derived from Hemangioblastoma Tumor Cells (A) Giemsa staining of the vacuolated neoplastic stromal cell of the hemangioblastoma. (B) Giemsa staining of early (arrows), late (arrowheads), and dividing (open arrow) nucleated erythrocytes expanding in an erythropoietin-enriched medium. (C) Giemsa staining of immature (arrow) and mature (arrowhead) polynuclear granulocytes expanding in an erythropoietin-enriched medium. (D) While loss of heterozygosity was not detected in peripheral blood mononuclear cells (PB), deletion of the wild-type *VHL* allele occurred in cultured hemangioblastoma (T) and cultured nucleated erythrocytes (NE), confirming the hemangioblastic origin of the nucleated erythroid cells.

At concentrations of 50 U/ml of erythropoietin or more, hemangioblast cells were sustained and expanded in culture (Figure 4). The identity of the cultured hemangioblasts, which was confirmed by morphology and immunofluorescence, demonstrated coexpression of erythropoietin and erythropoietin receptor. At culture concentrations of erythropoietin at 100 U/ml, two other populations of nucleated cells (nonadherent and adherent) appeared. The nonadherent cells were identified by Giemsa staining as nucleated erythrocyte or granulocyte progenitors in various stages of differentiation (Figure 4). These hematopoietic progenitor cells expanded over two weeks when the expansion stopped and the absolute number of cells remained stable. Adherent cells were identified as endothelial cells morphologically (flat and elongated) and by expression of endothelial markers Tie-2 and CD31. These cells expanded beginning at two weeks and continued to expand while in culture (at least 90 d).

### Origin of Hematopoietic and Endothelial Progeny

To determine their origin, *VHL* gene deletion analyses were performed on microdissected portions of uncultured tumors, cultured tumor cells, cultured hematopoietic progeny, and cultured endothelial progeny. Deletion of the wild-type *VHL* allele (loss of heterozygosity) occurred in the uncultured tumor specimens, cultured tumor cells, cultured hematopoietic progeny, and cultured endothelial progeny, confirming their neoplastic origin (Figure 4). Peripheral blood–derived mononuclear cells from patients served as a negative control (Figure 4).

### Development and Expansion of Mature Hematopoietic Progeny

To determine if the tumor-derived nonadherent immature hematopoietic progenitors could be expanded into mature erythrocytic or granulocytic progeny, we developed a culture environment designed to expand immature hematopoietic cells ex vivo that exploited the characterized receptor expression (erythropoietin, IL3, and c-kit). In this environment, expansion of mature enucleated erythrocytes and granulocytes occurred by day 3 with maximal proliferation occurring by day 7. In vitro senescence of mature hematologic progeny occurred beyond day 7. The mature erythrocytes displayed characteristics similar to that of normal erythrocytes, including a mean cell volume of 80.5 fl, a mean cell hemoglobin of 29.7 pg, and a mean corpuscular hemoglobin concentration of 37 g/dl. The mature granulocytes displayed characteristic morphology and the presence of CD13 mRNA by RT-PCR.

### Expansion of Endothelial Progeny

**Figure 5:**
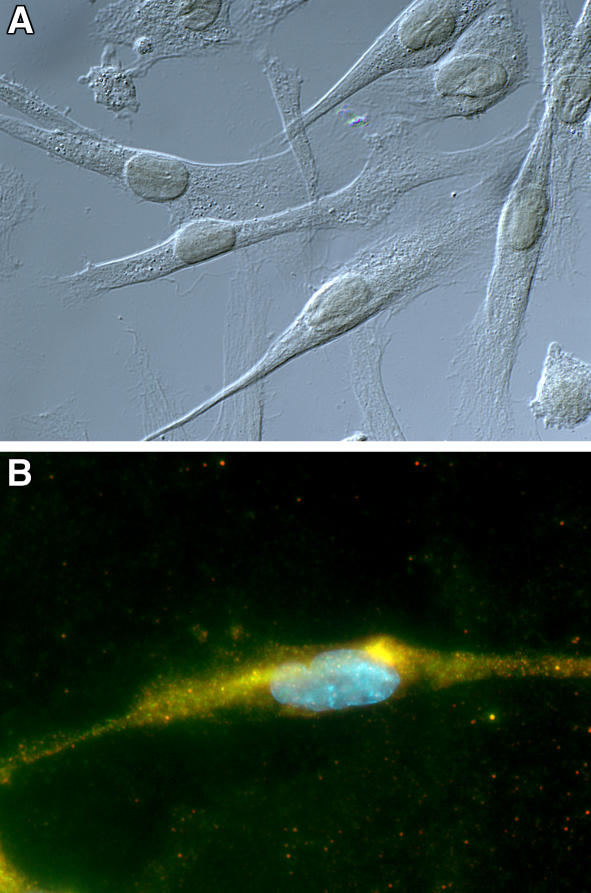
Endothelial Progeny Derive from Hemangioblastoma Tumor Cells (A) Differential interference contrast image of adherent cells derived from cultured hemangioblasts. Note the flat, elongated morphology similar to endothelial cells. (B) These cells expressed the endothelial markers Tie-2 (red), and CD31 (green) (nuclear stain 4′,6-diamidino-2-phenylindole; blue).

To expand the tumor-derived immature endothelial progeny, we used a culture media designed to expand immature endothelial cells ex vivo that exploited the characterized receptor expression (erythropoietin, IL3, c-kit, and Flk-1 [VEGF receptor-2]). The adherent endothelial cells continued to proliferate while in culture (at least 30 d). These expanded cells were adherent and morphologically similar to endothelial cells (flat and elongated). Consistent with an endothelial lineage, these cells expressed the markers Tie-2 and CD31 (Figure 5).

## Discussion

Identification of the cell of origin in tumors will improve understanding of their pathobiology and treatment. For familial tumor suppressor syndromes, it may also explain the unique tissue distribution of tumor involvement. We found that the neoplastic cells of origin in CNS hemangioblastomas from VHL patients are derived from embryologically arrested mesoderm cells and are hemangioblasts with hematopoietic and vasculogenic (endothelial cell) potential. These cells retain their multipotent differentiation ability and are able to develop into hematopoietic and endothelial progenies. The identification of the cell of origin of this tumor enhances understanding of the biological basis and tumor distribution of this familial tumor suppressor syndrome and may provide therapeutic opportunities.

### Cell of Origin for Hemangioblastoma

From analysis of chick embryo development, Sabin (1920) hypothesized the existence of a multipotent embryologic precursor cell capable of forming both blood and vessels [18]. Based on Sabin's hypothesis and the discovery of intratumoral blood and vessel formation in CNS hemangioblastomas, Stein et al. (1960) suggested that an arrested angiomesenchymal cell or embryologic defect was the origin of these tumors [12]. Based on embryonic stem cell lineage studies, Choi et al. (1998) discovered the multipotent embryonic precursor for both hematopoietic and endothelial cells that Sabin had hypothesized, and defined it as a “hemangioblast” [17]. Despite these observations, the histogenesis of hemangioblastomas has been unclear. It was only recently that Vortmeyer et al. (1997) identified the stromal cell in hemangioblastomas as the underlying neoplastic cell [19]. These previous findings and recent insights into the molecular basis of embryologic development [16,20,21] and tumor formation now permit investigation into the origin and multipotent capability of the neoplastic cells in hemangioblastomas.

To investigate whether the cell of origin in hemangioblastomas is the embryologically arrested hemangioblast, we characterized the neoplastic stromal cells from CNS hemangioblastomas derived from VHL patients. Consistent with a cell of mesodermal origin, the neoplastic stromal cells in these hemangioblastomas expressed the protein brachyury [16]. Expression of brachyury, Flk-1 (VEGF receptor 2), and Scl in the neoplastic stromal cells confirm that the tumor cell of origin in hemangioblastomas is an embryologic hemangioblast [20]. Expression of Scl confirms that these are hematopoietic stem cells with self-renewal capabilities and that they are committed to hematopoietic and endothelial lineages [22–26]. These findings also indicate that CNS hemangioblastomas are derived from committed hematopoietic precursors and not a neurogenic progenitor, as has been described with other primary CNS malignancies [27,28].

Because brachyury is expressed only during early mesoderm development [16], its expression in these neoplastic cells suggests that VHL-related hemangioblastomas originate from embryologically arrested mesodermal cells. This finding has several potential implications for hemangioblastoma development in VHL. First, it suggests that the precursor cells (hemangioblasts) for hemangioblastoma formation are present during embryologic development. Second, it also implies that loss of the wild-type allele, which is necessary for hemangioblastoma formation in VHL [29], occurs during embryogenesis. Third, these observations indicate that *VHL* tumor suppressor gene function affects development. These findings are consistent with previous studies that demonstrate that loss of tumor suppressor gene function can affect normal tissue development [30–32]. Recently, Zhu et al. [33] found that mice genetically engineered to lack the *neurofibromatosis-1* tumor suppressor gene demonstrated a variety of developmental defects that resulted in increased proliferation of glial progenitors cells, which in some cases led to optic nerve glioma formation (part of the neurofibromatosis-1 neoplasia phenotype). Fourth, an embryologically arrested hemangioblast may explain the presence of fetal hemoglobin within blood islands of resected hemangioblastomas from VHL patients [13,34]. Finally, these data support an emerging concept of tumor formation during development in a variety of different tumor types. Specifically, Trichopoulos et al. [35] has hypothesized a perinatal site-specific increase in stem cells as an underlying mechanism of breast cancer development and Samuelson et al. [36] found that increased head circumference was positively correlated with the incidence of childhood brain cancer, implying that pathogenesis occurs during fetal life.

### Development of Hematopoietic and Endothelial Progeny

To determine if the hemangioblasts in hemangioblastomas have the same multipotent developmental capacity (hematopoietic and endothelial) as hemangioblasts during embryologic development, we further characterized these cells and analyzed their capacity for multilineage differentiation. Tumors were characterized by scattered expression of hematopoietic stem cell markers CD133 and CD34, as well as stem cell factor receptor (c-kit) and IL-3 receptor. Based on the uniform expression of erythropoietin and erythropoietin receptor by the hemangioblasts, suggesting a neoplastic erythropoietin-driven autocrine loop [13,37], we initially cultured the neoplastic cells in an erythropoietin-enriched media. Similar to embryologic hemangioblasts, hemangioblastoma-derived hemangioblasts demonstrated self-renewal and differentiated into hematopoietic (erythroid and myeloid) and endothelial lineages in this environment. These findings are in agreement with observations in other tumors in which stem cells have been proposed as the origin of tumor initiation and propagation [27,38–41].

According to Knudson's “two-hit” hypothesis of tumorigenesis [42], initiation of tumor formation follows inactivation of both alleles of the *VHL* gene. Because germline mutations of the *VHL* gene were present in all of these patients. and deletion of the remaining wild-type allele is necessary to develop VHL-associated tumors (i.e., hemangioblastomas), we were able to confirm the origin of the hematopoietic and endothelial progeny by loss of heterozygosity analyses. Deletion of the wild-type *VHL* allele (loss of heterozygosity) was found in the cultured hematopoietic and endothelial progeny, confirming their neoplastic origin.

The functional ability of the hemangioblast-derived immature hematopoietic progenitors to expand into mature erythrocytic and granulocytic progeny was tested using a culture media designed to expand immature hematopoietic cells ex vivo [14] and to exploit the characterized receptor expression (erythropoietin receptor, c-kit, and IL-3). The capacity of the hemangioblast-derived endothelial progenitors to proliferate in a manner similar to early vasculogenesis was examined using a different culture medium designed to generate endothelial cells ex vivo and that also exploited the characterized receptor expression (erythropoietin receptor and Flk-1 [VEGF receptor-2]) [17]. As occurs with extramedullary hematopoiesis and blood island formation, expansion of mature enucleated erythrocytes and endothelial cells occurred in these environments. These findings are consistent with the presence of a developmentally arrested hemangioblast that can be functionally reactivated under appropriate environmental conditions and concur with histological evidence of intratumoral blood island formation in hemangioblastomas [12,13].

### Implications for Clinical Development of Hemangioblastomas

While our findings indicate that the cell of origin for VHL-associated hemangioblastomas are established during development, clinically significant or radiographic-evident hemangioblastomas rarely occur in infancy or early childhood in VHL. This observation is consistent with the known tendency of these tumors for slow growth and well-documented quiescent growth periods that can last for years [11,43]. Thus, it is likely that in early childhood many of these tumors remain asymptomatic and below the level of radiographic detection (even by sensitive MRI techniques). An analogous situation is the presence of endolymphatic sac tumors, which are undetectable by imaging for years in many patients with VHL [5,6,44].

The presence of subclinical and infraradiologic hemangioblastoma tumorlets in VHL patients at autopsy supports the concept that these tumors may be developmentally arrested but reactivated under appropriate conditions later in life [34]. There are several factors that could conceivably be involved in the propagation of these tumors. Circulating factors, including hormones (e.g., during puberty or pregnancy) could promote the growth of previously transformed tumor cells [45–47]. Similarly, local microenvironmental stimuli driven by autocrine or paracrine loops have been hypothesized to spur hemangioblastoma growth [13]. Finally, additional genetic alterations could underlie the observed delayed appearance and erratic growth of these tumors.

### Implications for the Distribution of Hemangioblastomas

Because embryologic development results in the precise topographical arrangement of various cell types, a hemangioblast-defined origin of hemangioblastomas could explain the distribution of these tumors. We recently examined the natural history and location of CNS hemangioblastomas (655 CNS hemangioblastomas in 160 consecutive patients) in VHL patients as defined by MRI [11]. Consistent with the distribution of hemangioblastomas found here, that study revealed that hemangioblastomas were found only within very defined regions of the CNS, including the cerebellum (38%), brainstem (10%), and spinal cord (51%).

This tumor distribution coincides with previous topographical analyses, which revealed that Scl is transiently expressed by embryologic hemangioblast cells during development in the retina, diencephalon, mesencepahlon, metencephalon, and spinal cord, but not the telencephalon [22,24,48–50]. Thus, the highly specific distribution of *Scl* gene expression during embryogenesis correlates with the restricted distribution of hemangioblastomas in VHL patients [11]. The rare occurrence (less than 1%) of hemangioblastomas in the supratentorial cerebral hemispheres may represent an ectopic rest of hemangioblasts. Taken together, these findings suggest that hemangioblastoma distribution in VHL does not result from migration of the tumor-initiating cells (hemangioblast); instead, developmental processes may govern distribution. The topographic and tissue-restricted distribution patterns of tumors that occur in other familial neoplasia syndromes may also arise by a similar mechanism.

### Study Limitations

Because we examined only VHL-associated hemangioblastomas, the results of this study may not be applicable to hemangioblastomas that arise sporadically. Moreover, because hemangioblastomas are benign neoplasms, these findings may not apply to malignant tumors, particularly those that do not arise in the context of tumor suppressor syndromes. Future analyses of other malignant tumors may be necessary to understand the factors involved with growth initiation, clinical development, and the developmental effects of tumor suppressor genes.

### Potential Clinical Implications

Several findings of this study may have future implications for clinical practice. The data here support the concept of a multipotent tumor stem cell as the initiating and sustaining cell in hemangioblastomas in the context of VHL. Specifically, an embryologically arrested hemangioblast that retains the ability to form blood and endothelial cells appears to underlie the formation and propagation of benign VHL-associated hemangioblastomas. Identification of the hemangioblast as the cell of origin in hemangioblastomas could permit the selective targeting of uniquely expressed proteins or modulation of specific molecular pathways in the selective treatment of these neoplasms. Moreover, strategies that exploit the known differentiation potential (blood and endothelial cells) of the hemangioblast may offer an alternative treatment paradigm.

### Conclusions

These findings indicate that the neoplastic cells from CNS hemangioblastomas from VHL patients are hemangioblasts derived from embryologically arrested mesoderm. These embryologic tumor cells retain their multipotent differentiation ability and can develop into hematopoietic or endothelial progeny. The identification of the cell of origin of this tumor enhances understanding of the biological basis of this familial tumor suppressor syndrome and may provide therapeutic opportunities.

## Supporting Information

### Accession Numbers

The GenBank (http://www.ncbi.nlm.nih.gov) Entrez Gene identification number for *VHL* is 7428.

## Abbreviations

CNS: central nervous system
IL: interleukin
MRI: magnetic resonance imaging
RT-PCR: reverse transcription PCR
VEGF: vascular endothelial growth factor
VHL: von Hippel-Lindau disease
